# A Novel Gene Signature for Molecular Diagnosis of Human Prostate Cancer by RT-qPCR

**DOI:** 10.1371/journal.pone.0003617

**Published:** 2008-10-31

**Authors:** Federica Rizzi, Lucia Belloni, Pellegrino Crafa, Mirca Lazzaretti, Daniel Remondini, Stefania Ferretti, Piero Cortellini, Arnaldo Corti, Saverio Bettuzzi

**Affiliations:** 1 Department of Medicina Sperimentale, University of Parma, Parma, Italy; 2 Istituto Nazionale Biostrutture e Biosistemi (I.N.B.B.), Roma, Italy; 3 Department of Patologia e Medicina di laboratorio, University of Parma, Parma, Italy; 4 Dimorfipa, University of Bologna, Bologna, Italy; 5 Urology Operative Unit, Azienda Ospedaliera-Universitaria of Parma, Parma, Italy; 6 Department of Scienze Biomediche,University of Modena, Modena, Italy; Memorial Sloan-Kettering Cancer Center, United States of America

## Abstract

**Background:**

Prostate cancer (CaP) is one of the most relevant causes of cancer death in Western Countries. Although detection of CaP at early curable stage is highly desirable, actual screening methods present limitations and new molecular approaches are needed. Gene expression analysis increases our knowledge about the biology of CaP and may render novel molecular tools, but the identification of accurate biomarkers for reliable molecular diagnosis is a real challenge. We describe here the diagnostic power of a novel 8-genes signature: ornithine decarboxylase (*ODC*), ornithine decarboxylase antizyme (*OAZ*), adenosylmethionine decarboxylase (*AdoMetDC*), spermidine/spermine N(1)-acetyltransferase (*SSAT*), histone H3 (*H3*), growth arrest specific gene (*GAS1*), glyceraldehyde 3-phosphate dehydrogenase (*GAPDH*) and Clusterin (*CLU*) in tumour detection/classification of human CaP.

**Methodology/Principal Findings:**

The 8-gene signature was detected by retrotranscription real-time quantitative PCR (RT-qPCR) in frozen prostate surgical specimens obtained from 41 patients diagnosed with CaP and recommended to undergo radical prostatectomy (RP). No therapy was given to patients at any time before RP. The bio-bank used for the study consisted of 66 specimens: 44 were benign-CaP paired from the same patient. Thirty-five were classified as benign and 31 as CaP after final pathological examination. Only molecular data were used for classification of specimens. The Nearest Neighbour (NN) classifier was used in order to discriminate CaP from benign tissue. Validation of final results was obtained with 10-fold crossvalidation procedure. CaP versus benign specimens were discriminated with (80±5)% accuracy, (81±6)% sensitivity and (78±7)% specificity. The method also correctly classified 71% of patients with Gleason score<7 versus ≥7, an important predictor of final outcome.

**Conclusions/Significance:**

The method showed high sensitivity in a collection of specimens in which a significant portion of the total (13/31, equal to 42%) was considered CaP on the basis of having less than 15% of cancer cells. This result supports the notion of the “cancer field effect”, in which transformed cells extend beyond morphologically evident tumour. The molecular diagnosis method here described is objective and less subjected to human error. Although further confirmations are needed, this method posses the potential to enhance conventional diagnosis.

## Introduction

Prostate cancer (CaP) is believed to become the most relevant cause of cancer death in the Western Countries in the near future because its incidence increases rapidly with age. Since prognosis is generally unfavourable when disease is no longer organ-confined, detection of CaP at an early curable stage is highly desirable, but unfortunately available methods such as serum Prostate Specific Antigen (PSA) present limitations [1]. The diagnosis of CaP is conventionally obtained by saturation prostate biopsy and morphological examination of tissue sections. This method is reliable, but requires careful training. Nevertheless, intra- and inter-observer incongruities may occur. In principle, molecular diagnosis would be more objective and, hopefully, significantly less subjected to human error if obtained with reliable methods. But the ideal method should also be fast, standardized and economically convenient. Such achievement is indeed possible in theory, but results published are not completely satisfactory yet, also because they are usually based on a conventional approach with takes advantage of a single molecular predictor significantly up- or down-regulated in the cancer specimen versus benign control. A major obstacle to this goal is that the molecular events causing CaP onset and progression are still far from being completely revealed: very few well known oncogenes or tumor-suppressors have been clearly linked to prostate tumorigenesis, and for this reason CaP is still considered an elusive disease. Therefore, new molecular approaches for early screening and diagnosis are urgently needed.

Gene expression analysis was recently used to increase our knowledge about the biology of CaP [2]. Gene signatures at RNA level are determined and often used as predictors to model clinically relevant information (e.g. prognosis, survival time, sensitivity to drugs, etc.). To this aim, final conclusions on the classification power of the gene signature studied are entirely drawn on the basis of the molecular data obtained at transcriptional level by using methods such as DNA microarray or RT q-PCR. By Northern blot analysis, we have previously identified 8 informative genes whose expression changes on the basis of the presence of CaP malignancy in humans [3]. In a 5-year follow-up study, we also showed that the levels of expression of this panel of genes strongly correlate with differentiation and final outcome (prognosis) of CaP patients. This result was obtained by combining molecular data with standard clinical information [4]. But in our mind the real challenge was to detect CaP by means of molecular tools alone. Although the study of specifically altered gene expression during CaP tumourigenesis is a difficult task, the scientific information that will ultimately be obtained is an important reward, leading to a better understanding of the molecular basis of the disease which may lead to better methods for diagnosis and therapy. This is particularly important when considering the heterogeneity of CaP and the variability of clinical progression, with some patients presenting with slow growing indolent tumours and other patients having a disease that is rapidly progressive.

The signature that we have identified is made of 4 metabolically related genes, *ODC*, *OAZ*, *AdoMetDC*, *SSAT*, the regulatory genes of the polyamine metabolism, 2 genes related to cell cycle progression, *H3* and *GAS1*, specific markers of S- and G_0_-phases, respectively [5], [6], *GAPDH*, an enzyme of the glycolitic pathway, and *CLU* an enigmatic protein whose biological role is still a matter of debate.

*CLU*, also known as *SGP-2*, *TRPM-2* or *ApoJ*, is highly over-expressed during prostate gland involution and remodelling [7], [8]. Although there is a general consensus about the involvement of CLU in regulating cell death, its specific role in the apoptotic process is still controversial, as well as in cell transformation. More specifically, its level of expression and regulation during CaP onset and progression is debated [9], [10]. A better understanding of this issue is of particular importance not only because of the above considerations, but also because a clinical trial aimed at silencing *CLU* gene by antisense oligonucleotides in CaP patients is currently ongoing [11]. As a matter of fact, we and others have found that *CLU* is down-regulated during CaP progression [12]–[15], suggesting that it might act as a tumour-suppressor factor [16], [17].

Concerning the gene signature described here, all of the genes studied have previously shown strictly related shifts in co-expression during CaP progression [3]. Specific alteration of transcription of the regulatory enzymes of polyamine metabolism during CaP progression has also been validated by meta-analysis of microarrays [18]. In the TRAMP mice model of CaP progression, our gene signature alone as determined by RT-qPCR, besides discriminating CaP from benign tissue, also predicted individual response to treatment with Green Tea Catechins (GTCs) [19]. Other individual marker genes have been found of proven validity in this field, such as α-methylacyl coenzyme A racemase (*AMACR*) [20], [21] or Prostate Cancer Antigen 3 (*DD3/PCA3*) [22], but at the moment no relationship between the expression level of PCA3 and tumour grade or staging was found yet. Our gene signature is made of informative genes some of which are well known to play important roles in the physiology of prostate cells, such as the genes coding for regulatory proteins of polyamine metabolism [23] and *CLU* [7].

The aim of our study was to show that our gene signature alone enhances the sensitivity and the specificity of molecular diagnosis of CaP when compared to single marker identification. We used histopathologic classification of fixed tissues specimens as final reference, as usually done in similar circumstances [24], [25]. The 8-gene model was detected by RT-qPCR in frozen tissue specimens obtained at Radical Prostatectomy (RP). Classification of tissue specimens (i.e. presence or absence of tumour) was performed without other pathological or clinical data. Furthermore, molecular data were used for sub-classification of tissue specimens with regard to Gleason score, age and total serum PSA of the patient at RP.

For specimen classification we used the Nearest Neighbour (NN) classifier, a statistical multi-factorial analysis tool known to perform well specifically for cancer classification when compared with other methods. For validation of classification performance, we used the 10-fold cross validation procedure, repeated 100 times with different sub-samplings in order to estimate the mean performance of the signature and the confidence interval (results are expressed as mean±95% confidence interval, approximately corresponding to 2 standard deviations).

## Results

### The tissue specimens bio-bank

The bio-bank consisted of a collection of 66 human specimens, see Table 1, matching our eligibility criteria for the study: i) pathological evidence of presence or absence of CaP in the frozen pre- and post-RNA sections; ii) benign specimens free of prostate intraepithelial neoplasia (PIN) lesions or tumour invasion, taken very far away (i.e. in different areas of the prostate, ideally in the controlateral sextant) from the neoplastic lesion; iii) CaP specimens having a cancer cell content covering at least 5% of the whole section area; iv) good yield and high quality of RNA preparation. Among these, 44 specimens were CaP-benign paired specimens obtained from the same patient. Thirthy-five specimens were classified as benign by the pathologist, while 31 were CaP.

**Table 1:** complete list of clinical cases with clinical data at radical RP and final pathological or molecular classification with Leave-One-Out cross validation procedure in order to obtain a unique classification for each sample.

Patient #	Age	PSA_tot_ (ng/ml)	Gleason score (whole gland)	Pathological diagnosis of tissue specimen 1:	Molecular diagnosis tissue specimen 1:	Pathological diagnosis of tissue specimen 2:	Molecular diagnosis tissue specimen 2:
1	68	2,8	G2 score4	BENIGN	BENIGN		
2	66	4,72	G3 score5	BENIGN	BENIGN		
3	62	3,07		BENIGN	BENIGN		
4	76	5,74	G3 score6	BENIGN	BENIGN	CaP 15%	CaP
5	72	11,9		BENIGN	BENIGN	CaP 80%	CaP
6	69	38,82		BENIGN	*CaP*	CaP 25%	*BENIGN*
7	61	6,32		CaP 50%	CaP	CaP 5%	CaP
8	68	6,90		BENIGN	BENIGN	CaP 40%	*BENIGN*
9	67	10,8		BENIGN	BENIGN		
10	64	4,42		BENIGN	BENIGN		
11	73	4,28		BENIGN	BENIGN		
12	72	3,72		BENIGN	BENIGN		
13	64	4,21		BENIGN	BENIGN		
14	71	6,39		BENIGN	*CaP*	CaP 65%	CaP
15	68	8,53		BENIGN	BENIGN	CaP 15%	CaP
16	75	8,16		BENIGN	BENIGN	CaP 5%	*BENIGN*
17	64	4,2	G3 score7	BENIGN	BENIGN		
18	59	15,07	G4 score7	BENIGN	BENIGN	CaP 25%	CaP
19	67	49,30		BENIGN	BENIGN	CaP 5%	CaP
20	65	19,70		BENIGN	BENIGN	CaP 90%	CaP
21	68	9,75		BENIGN	BENIGN	CaP 25%	CaP
22	67	9,1		CaP 5%	CaP	CaP 90%	CaP
23	74	12,1		CaP 25%	CaP	CaP 40%	CaP
24	68	12		BENIGN	BENIGN	CaP 90%	CaP
25	64	5,97		BENIGN	*CaP*		
26	71	9,04		BENIGN	BENIGN		
27	61	9,6		CaP 85%	CaP		
28	77	17,7		CaP 90%	CaP		
29	70	11,26		BENIGN	*CaP*	CaP 5%	CaP
30	62	4,35		BENIGN	BENIGN		
31	72	4,5		BENIGN	BENIGN	CaP 10%	*BENIGN*
32	72	6,55	G4 score8	BENIGN	BENIGN	CaP 20%	CaP
33	60	11,7		BENIGN	BENIGN		
34	71	5,6		BENIGN	*CaP*	CaP 5%	CaP
35	53	11,10	G5 score8	BENIGN	BENIGN	CaP 55%	CaP
36	65	5,04		BENIGN	BENIGN	CaP15%	CaP
37	66	5,2		BENIGN	*CaP*	CaP 5%	CaP
38	71	16,8		BENIGN	*CaP*	CaP 30%	CaP
39	70	4,68		CaP 15%	CaP		
40	65	7,78	G5score9	BENIGN	BENIGN	CaP 10%	*BENIGN*
41	72	10,8		BENIGN	BENIGN	CaP 85%	CaP

Mean age of the patients was 66.8±5.7. Mean PSA value at RP was 10.2±8.7 ng/mL. Gleason score is given by examination of the whole gland (fixed and embedded) after RP. Pathological evaluation of the frozen sections is indicated, together with percent of cancer cells given as a mean of pre- and post-RNA frozen sections. 44/66 were CaP-benign paired specimens obtained from the same patient. Specimens misclassified by the molecular method are in bold italic.

### How to get molecular and morphological data from the same tissue specimen: the “sandwich” method

**Figure 1:**
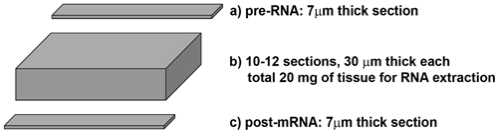
Sandwich procedure developed for collection of specimens and direct comparison of morphological and molecular classification.

To obtain a direct comparison between molecular data and pathological classification we developed a “sandwich” method (see Figure 1 and Methods: Prostate Tissue Specimens Collection and Handling). RNA extraction yielded an average of 50–60 µg of total RNA from 20 mg of human prostate tissue. The amount of total RNA obtained was high enough to directly check the quality of the preparation by spectrophotometry, followed by conventional electrophoresis. Only good quality RNA was used for RT-qPCR analysis. Eight informative genes plus 2 housekeepers were analysed starting from the same RNA sample.

### RT q-PCR and data analysis

**Figure 2:**
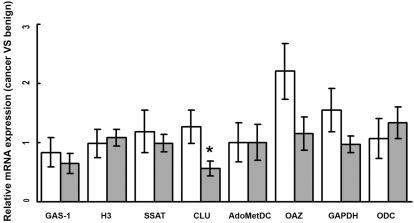
Relative gene expression (cancer versus benign) as a function of RP Gleason score. * p value<0.01 (t-test). White bars = Gleason score<7; Grey bars = Gleason score≥7. Error bars represent the standard error of the mean.

Relative quantification of the target genes was performed with the well known REST software tool [26]. Changes in the level of expression of 7 out of 8 informative genes in CaP Versus benign tissue did not reach statistical significance (not shown), while *CLU* was significantly down-regulated in CaP (p<0.05). In Figure 2, the relative gene expression of the signature obtained by the 2^∧−ΔΔCT^ method is reported as a function of RP final Gleason score. Interestingly, *CLU* is significantly and reversely related (p<0.01) to the Gleason score of the tumour: i.e. lower expression levels of *CLU* were found in higher Gleason score specimens. In Table 2 are shown the final results of classification using the Nearest Neighbour (NN) classifier combined with the 10-fold cross validation. Although the REST analysis showed that only the changes in *CLU* expression were statistically significant, NN classification+10-fold cross validation performed on ΔCt data revealed a very good performance, in that the discrimination of CaP versus benign specimens was obtained with a combination of 7 genes (*H3, GAS1, SSAT, CLU, AdoMetDC, OAZ, ODC*), using 3 neighbours and the correlation-based distance. This classification is statistically significant.

**Table 2:** Final results of classification using the Nearest Neighbour (NN) classifier.

DICHOTOMIES DISCRIMINATED	% CORRECT CLASSIFICATION
CaP vs Benign	(80±5)
Age: ≤65 vs >65	84
Gleason score: <7 vs ≥7	71
PSA_tot_ (ng/mL): <10 vs ≥10 ng/mL	42

The 10-fold cross validation procedure was used in order to estimate the mean performance of the signature and the confidence interval. CaP versus benign specimens were discriminated with (80±5)% accuracy, (81±6)% sensitivity and (78±7)% specificity. The result is expressed as mean±95% confidence interval, approximately corresponding to 2 standard deviations.

In this work we paid the highest attention to realize the best possible quality of tissue sampling and characterization (Figure 1). This is absolutely required giving the high heterogeneity of a prostate cancer specimen. The combination of a “robust” and widely tested approach for statistical analysis of data with high quality specimen sampling allowed us to obtain the final validated result with a dataset of 66 specimens.

The concordance between our gene signature classification and final pathological classification (CaP versus benign) obtained by the pathologist was (80±5)%, with a sensitivity of (81±6)% and a specificity of (78±7)%. Tissue specimens that have been misclassified by the molecular method, with respect to pathological classification, are indicated in Table 1 (bold). We remark that in this case a Leave-One-Out cross validation has been considered, since with the N-fold procedure misclassifications of single samples may vary at each realization. With the same combination of genes we correctly sub-classified 84% of tumour specimens with regard of patient's age, 71% with regard to final RP Gleason score and 42% with regard to PSA before RP (Table 2). The same signature did not perform well for classification with regard to TNM staging.

### Inter-laboratory validation methodology

A second laboratory was participating in an inter-lab validation methodology. Each tissue specimen was analyzed individually, determinations were in duplicate wells and each experiment was run for 6 times in both independent laboratories, with Ct line set at the same value. Inter-laboratory variability was less than 1 cycle for each single determination. (data not shown). Such variability did not affect significantly final classification.

### As little as 1 mg of cancer tissue within 20 mg of benign specimen was detected

As shown in Table 1, the amount of cancer tissue was only 5% in 7/31 specimens (equal to 22.5% of total specimens; 6/7 have been correctly classified), and less than 15% in 13/31 specimens (equal to 42% of total specimens; 10/13 have been correctly classified). Therefore, the relative cancer tissue component accounted for only about 1–3 mg of tissue out of 20 mg total. Table 1 provides a complete list of the clinical cases from which each tissue specimen was obtained, also including clinical data, classification by the pathologist and percent of cancer cells present in the specimen, expressed as a mean value between the pre- and the post-RNA sections (see Figure 1).

## Discussion

### Identification of novel and accurate molecular biomarkers: the challenge

The identification of novel and accurate biomarkers for reliable molecular diagnosis of CaP is a real challenge. Previous studies by DNA microarray have identified molecular signatures that significantly correlated with CaP progression [27] or grading [28], leading to identification of informative genes (sometimes with unknown functions) that are still awaiting further studies for elucidating their role in CaP progression [29]. Other successful studies resulted in molecular diagnosis of CaP, but they were only based on few selected, cancer-specific genes. PCA3 is the best single CaP predictor: it is highly specific, but the sensitivity is relatively low [30]. Moreover, the sensitivity did not improve even when two more prostate-related genes were added [24], [25].

### A novel and reliable method based on a robust approach

Our work provides a reliable method for the molecular diagnosis of CaP that takes advantage of a relatively simple method. The 7-gene model here presented is based on the determination of genes which are known to be expressed in both benign and cancer tissue, and subjected to high individual variation. Therefore, the novelty of our approach consist in achieving efficient molecular classification of CaP with regard to final pathological diagnosis without using genes that are specifically highly up- or down-regulated in benign versus cancer tissue. In fact, our work is based on the detection and analysis of the expression of genes which are long time known to be physiologically expressed as well as essential for the biology of prostate cells. For instance, aliphatic polyamines are produced at very high levels by prostate epithelial cells and then released in prostate fluid in mM amounts [31]–[33], although the precise role of these compounds is still unknown. Detection of polyamines in urine was already attempted long time ago for the diagnosis of CaP [34].

To achieve a reliable detection of our gene signature in cancer tissue specimens and to implement practical use we had to address several technical issues. In absence of any dedicated specific tool or instrument, the development of a “sandwich” procedure was necessary for obtaining morphological and molecular data from the same tissue specimens (Figure 1).

### Statistical analysis

In previous publications, working with both animal [14] and human [3], [4] CaP models we have demonstrated strictly related shifts in the expression of all genes studied. Nevertheless, the REST analysis showed that the relative expression of 7 genes besides CLU did not reach statistical significance in malignancy. This was somehow unexpected. The difficulty of pursuing a molecular approach to CaP diagnosis in the real clinical setting is evident. But this result is explained by the fact that polyamine genes expression are known to be characterized by high individual variation in the tissue specimens analysed. Nevertheless, this did not jeopardize our analysis, because the molecular classification of tissue specimens here presented takes into consideration the whole gene signature, detecting a significant difference in the entire gene expression profile regardless of individual variations. The final result is that the integrated pattern of expression is significantly different in CaP versus benign controls. This novel approach has been successful using the NN classifier, a statistical method known to perform well specifically for cancer classification even when compared with other sophisticated methods [35], [36]. NN is a nonparametric method that belongs to the class of “robust” classifiers [37]. NN is well known to render a good performance where the boundary between the two classes to be discriminated is not clear-cut from the geometric point of view. This condition often happens in clinical studies in which individual variations may be much higher as compared to laboratory samples obtained from *in vitro* experiments, providing “fuzzy” boundaries which might result from complex gene expression profiles. NN is virtually not sensible to limited sampling of the classes to be discriminated because of the very limited number of parameters (namely, the choice of the distance function and the number of neighbours). Nevertheless, NN renders very good classification performances also compared to other multiparametric well known classifiers like Neural Networks or Support Vector Machines, methods that achieve optimal performance only when large datasets are available.

To date, the best approach to reach a final evaluation on the performance of even a “robust” statistical classifier such as NN is to reuse the collected data both for the training and the validation of the chosen classifier: this procedure is commonly referred to as cross validation (well described in many "classical" statistical books [37]). In N-fold cross validation, the dataset is randomly divided into N parts and each part is used as a validation set using the other as training set. Under these working conditions the classifier performance varies for each random realization. We applied this procedure both with a 5-fold (not shown) and a 10-fold cross validation, and obtained similar performances. Because of the above considerations, the result obtained by NN+10-fold cross validation procedure can be considered as very reliable even with a small-size dataset. The same statistical approach has been used already to analyze and validate gene expression signatures in cancer research [38], [39].

Under these conditions, the best classification performance was obtained with a 7-gene model (*H3*, *GAS1*, *SSAT*, *CLU*, *AdoMetDC*, *OAZ* and *ODC*). The concordance between molecular and final pathological classification had an accuracy (80±5)% with a sensitivity of (81±6)% and a specificity of (78±7)%. This result demonstrates that diagnosis of CaP by molecular data alone is feasible.

### The gene signature detects very few cancer cells: the cancer field effect

Notably, our method showed a very high sensitivity working on a collection of CaP tissue specimens in which a significant portion of the total, namely 7/31, were considered CaP on the basis of having only 5% of cancer cells. This accounts for only 1 mg of cancer tissue present in a total of 20 mg. A rationale consequence of this result is that molecular events detected in pre-malignant tissue or in tissues adjacent to cancer have provided diagnostic information, supporting the hypothesis that a molecular approach for CaP diagnosis would be a more sensitive and powerful tool than morphological examination. This result is consistent with the “cancer field effect”, as recently hypothesized. According to this idea, transformed cells extend beyond morphologically evident tumour. These events would precede development of malignancy, because normal appearing prostate tissue can undergo genetic changes in response to, or in expectation of, morphologic cancer [40]. Recently, field effects based on epigenetic events [41], nuclear matrix alterations [42], androgen receptor immunoreactivity in the stroma surrounding cancer lesion [43] have been discovered. Using oligonucleotide microarrays, another confirmation derived by a study in which the expression profiles of primary prostate cancer, adjacent normal tissue and normal tissue from tumour free donors were compared [44]. This scenario reinforce the requirement for objective molecular biological markers of the aggressive phenotype to resolve uncertainties with respect to identification of those precursor lesions which are most likely to progress to invasive and metastatic prostate cancer. The most likely explanation for misclassification between molecular and morphological data obtained in our study is that very early molecular changes may precede alteration of morphology, and therefore these conditions would not overlap (or be associated with) the pathological response. Molecular changes preceding changes in phenotype would be detected by the molecular method for diagnosis, increasing its sensitivity and explaining why 7 specimens (Table 1, bold) were seen as cancer by the gene signature method, while the pathological classification was benign. In this regard, higher sensitivity of the method would result in reducing the number of samples that need to be taken, with a clear advantage for the patient. It is known that sensitivity of biopsy is a potential problem, explaining why saturation biopsies have to be taken from patients. The procedure of saturating the prostate with biopsies according to volume to maintain a constant density of probing was developed in order to improve CaP detection rate, and prostate biopsies are more and more often becoming saturation biopsies [45]. Saturation biopsy can be considered for patients at risk of cancer who are willing to accept the side effects, but it is known that clinically insignificant cancers can be detected [46].

On the other hand, the same rationale might also explain the opposite result. In fact, the link between morphological lesions (i.e. HG-PIN) and clinical CaP is still a matter of debate [47], [48]. Also PIN represents a “field effect” with a potential for cancer progression, but may be not directly involved in or may not lead to the development of invasive prostate cancer [49]. Therefore we might hypothesize that certain changes in morphology might not compulsory be associated to clinically relevant cell transformation. Thus, specimens classified as benign by the molecular tool, but cancer instead by the pathologist because of altered morphology (5/31, Table 1, bold italic), might actually be different morphological entities or clinically indolent lesions, possibly undergoing regression because surrounded by reacting tissue. If so, this material would be not significant from the clinical point of view, but clearly distinguishable on a molecular basis from aggressive disease, although we have no definitive evidence of this at the moment.

### Clusterin and prostate cancer

*CLU* was cloned, sequenced and identified as the major up-regulated gene during massive induction of apoptosis and prostate regression caused by androgen depletion [7] or administration of Finasteride [50] and vitamin D analogues [51], [52]. The glycosylated, extracellular form of CLU is produced at high basal level by prostate cells and secreted in the prostate fluid, although its possible role in reproduction is still puzzling the researchers [53].

Authors have proposed that CLU might be over-expressed in cells surviving apoptosis, and not in cells doomed to die. Thus, a cytoprotective role for CLU has been proposed [54]. The debate on this issue is still wide open. It has been recently shown that different protein forms can be originated from the same gene by still unknown mechanisms [8]. TGF-beta and X-ray treatment induce a truncated form of CLU that localizes to the nucleus [55]–[58]. It is believed that different forms of CLU have different roles in human cells [8], also depending by their sub-cellular localization. Structural information concerning such protein forms are still scarce. Data concerning the possible involvement of *CLU* in transformation and tumour growth are still unclear or contradictory in the literature.

The REST relative expression analysis demonstrated (data not shown) that the down-regulation of *CLU* in CaP specimens is statistically significant (p = 0.023). This is consistent with our previous results [3], [13]. Although the specific issue of whether CLU is up- or down-regulated in CaP is still controversial [3], [13], [15], [59]–[61], confirmatory data supporting our hypothesis that CLU is not only down-regulated in CaP, but also in the most of cancers, can be easily retrieved from the Oncomine web site that collects data from more than 20,000 independent microarray experiments (http://www.oncomine.org). We have previously suggested that CLU might be a potential tumor-suppressor gene [51] acting through its nuclear form nCLU [57], [63], [64].

### Potential prognostic value of the method

We previously found that our informative genes have prognostic value [3], [4]. It is known that Gleason score is the best single predictor of CaP prognosis [65], but prediction of outcome in the 6–7 score range, comprising the most of clinical cases, is not satisfactory [65]. We had 28/41 of patients in these conditions in our bio-bank (Table 1). Unfortunately this analysis, as done before [4], requires definitive outcome data on the whole cohort of patient, and thus a minimum of a 5-year follow-up study will be necessary as previously done [4]. We are strongly encouraged to pursue this possibility because our informative genes have been already found of prognostic value not only in human CaP [4], but also in the TRAMP mice model. These animals were treated with Green Tea Catechins (GTCs) for inhibition of CaP progression [19]. In this experimental model, our gene signature was capable to efficiently discriminate CaP tissue specimens of TRAMP mice responding to GTCs treatment from those not-responding, therefore suggesting that the biological behaviour of CaP might be successfully investigated also in humans by the same approach.

In Table 2 it is also shown that the combination of 7 genes (*H3*, *GAS1*, *H3*, *SSAT*, *CLU*, *AdoMetDC*, *OAZ*, *ODC*) correctly classified 84% of cancer specimens with regard to age of the patient, 71% with regard to RP Gleason score and 42% with regard to PSA. Misclassification with regard to PSA was actually expected, because PSA is not a prostate tumour but, more properly, a prostate tissue specific marker. As a matter of fact, also PSA cut-off values and CaP detection rates are still a matter of debate [1]. Instead, we got a very good performance on classification of age and Gleason score, both widely used for clinical management of patients, with Gleason score being the best predictor [65]. Also these results strongly suggest that our approach is detecting important biological events occurring during prostate cell transformation and cancer progression.

For the above reasons it will be fundamental to continue this study by collecting clinical follow-up data, with a particular focus on misclassified cases, for re-classification of patients as a function of final outcome and possible validation of the potential prognostic power of the method.

### Final remarks

In conclusion, the RT-qPCR gene profiling method based on the gene signature described here appear to be an appropriate and reliable tool for molecular diagnosis of presence/absence of CaP not subjected to intra- and inter-observer error. This test could be of help to enhance diagnosis and particularly in those cases in which it is necessary to resolve possible uncertainties. Our approach, based on detection of a multiparametric expression signature, yielded the best performance at detection of CaP ever published with single predictors as far as we know. The method has potential prognostic value, because we previously demonstrated that the expression level of these genes correlate with the final outcome of CaP patients. Future research should be conducted to obtain the gene signature using less invasive means than a biopsy.

## Materials and Methods

### Prostate Tissue Specimens Collection/Handling and Pathological Classification:

Ethical approval for this work was granted by local Hospital Ethical Committee (Azienda Ospedaliera-Universitaria di Parma) and informed written consent was obtained from patients involved. Prostate surgical specimens were obtained from patients diagnosed with CaP by biopsy and recommended to undergo RP. No therapy was given to patients at any time before RP. Tissue specimens were excised out from the prostate gland immediately after surgery and quickly frozen in dry ice as previously described [3]. Two tissue specimens were obtained from the same prostate gland: one from the (supposed) cancerous portion of the gland, according to prostate mapping by biopsy. The second one from the (supposed) benign tissue taken very far away (i.e. in different areas of the prostate, ideally in the opposite sextant) from the neoplastic lesion. Both specimens were quickly frozen with dry ice to preserve RNA integrity, then embedded in OCT and frozen-sectioned. Representative serial sections (7 µm-thick) were E&H stained (Figure 1). Right after the last section (the so-called “pre-RNA” frozen section), 10–12 frozen sections at 30 µm-thickness, for a total of about 20 mg of tissue, were collected and stored at −80°C for RNA extraction. Then the cryostat was set back to 7 µm thickness and “post-RNA” frozen section were cut for E&H staining. This “sandwich” procedure (Figure 1) rendered 2 frozen sections with a thickness of 7 µm named “pre-RNA” and “post-RNA”. They were both used by the pathologist for the pathological classification of the 300–350 µm-thick tissue specimen contained in between. This material was then used for RNA extraction. Classification by the pathologist of the pre- and post-RNA sections was two-fold: presence or absence of cancer. No Gleason grade or score was given on these frozen sections because morphology was not preserved by fixation. Thank to this procedure we gained as much morphological information as possible from the tissue specimen that was homogenized for molecular analysis. For RT-qPCR analysis we only used CaP specimens with a cancer cell content covering at least 5% of the whole section area. Only specimens with no PIN lesions or tumour invasion were used as controls (benign). We also collected clinically relevant parameters: age of the patient, total serum PSA before surgery. Final Gleason score of the tumour was assessed after formalin fixation and pathological examination of the whole gland.

### RNA extraction

Total RNA was extracted from 20 mg snap-frozen human tissue specimens using RNA-fast (Molecular Systems, San Diego, CA) according to the manufacturer protocol. After spectrophotometric quantification, 2-µg aliquots were routinely electrophoresed on a 1% agarose gel to check quality and integrity as previously described [3].

### Retrotranscription

Two µg of total RNA from each sample was primed with 50 ng of random hexamers (Invitrogen) and incubated at 42°C for 60 min in a 30 µL (final volume) reaction mixture containing 50 mM tris HCl pH 8.3, 75 mM KCl, 3 mM MgCl2, 10 mM dithiothreitol, 0.5 mM dNTPs and 300 units of Superscript II Reverse Transcriptase (Invitrogen).

### Real-time PCR amplification

One µL of each cDNA preparation was PCR-amplified using the set of primers described below. PCR conditions were: 95°C for 15 min and then 95°C for 30 s, 60°C for 30 s and 72°C for 30 s repeated for 40 cycles, then 95°C for 30 s and 55°C for 30 s. Melting curves were obtained. RT-qPCR was performed on the DNA Engine Opticon 4 machine (MJ Research, Wathman, MA, USA) using a 2× SYBR Green customized master mix (Biodiversity s.r.l., Brescia, Italy).

Primers:

**GAS1** DIR: 5′- CGC TGA GCC GCT ACC TGA -3′
   REV: 5′- CTT GGG CAT AGC CAG CAT GT -3′
**H3** DIR: 5′- CAG GAG GCT TGT GAG GCC TA -3′
   REV: 5′- AGC TGG ATG TCT TTG GGC AT -3′
**SSAT** DIR: 5′ - GGT TGC AGA AGT GCC GAA AG -3′
   REV: 5′- GTA ACT TGC CAA TCC ACG GG -3′
**CLU** DIR: 5′- TGA TCC CAT CAC TGT GAC GG -3′
   REV: 5′- GCT TTT TGC GGT ATT CCT GC -3′
**ODC** DIR: 5′- AGA CCT TCG TGC AGG CAA TC -3′
   REV: 5′- AGG AAA GCC ACC GCC AAT AT -3′
**AdoMetDC** DIR: 5′- CAT CAC TCC AGA ACC AGA AT -3′
   REV: 5′- TAA CAA ACA AGG TGG TCA CA -3′
**OAZ** DIR: 5′- CCT CCA CTG CTG TAG TAA CC -3′
   REV: 5′- GAA AGA TTG TGA TCC CTC TG -3′
**GAPDH** DIR: 5′- AAC CTG CCA AAT ATG ATG AC -3′
   REV: 5′- TTG AAG TCA GAG GAG ACC AC -3′
**HMBS** DIR: 5′- TGA AAT CAT TGC TAT GTC CA -3′
   REV: 5′- ATG TTC AAG CTC CTT GGT AA -3′
**PGK1** DIR: 5′- CTT TCA TGT GGA GGA AGA AG -3′
   REV: 5′- TAG CTT GGA AAG TGA AGC TC -3′

### RT-qPCR data analysis

Analysis of the relative expression of target genes was performed with REST 2005 (Relative Expression Software Tool) BETA V1.9.9. [26]. Two housekeeper genes phosphoglycerate kinase 1 (*PGK1*) and hydroxy-methyl-bilane synthetase (*HMBS*) were used in the study. Also primers efficiency was calculated and used for REST analysis.

### Statistical Analysis

Raw Ct data were normalized and transformed in ΔCt by using the geometric mean of both housekeepers (*PGK1* and *HMBS*). Normalized ΔCt data were used for classification. The NN classifier was used in order to discriminate CaP from benign tissue. The 10-fold crossvalidation procedure was used for final validation in order to reduce the underestimation of the misclassification error due to over-fitting. All the possible combinations of the 8 genes, several numbers of neighbours and different distances between sample profiles (Euclidean distance, Correlation-based distance, Mahalanobis distance) were considered in the analysis. The same procedure was used for sub-classification of CaP specimens into several dichotomies: patients whose age was ≤65 versus >65; total PSA value at prostatectomy <10 versus ≥10; final Gleason score on fixed whole prostate gland <7 versus ≥7.

## References

[1] Pelzer AE, Tewari A, Bektic J, Berger AP, Frauscher (2005). Detection rates and biologic significance of prostate cancer with PSA less than 4.0 ng/mL: observation and clinical implications from Tyrol screening project.. Urology.

[2] Huppi K, Chandramouli G (2004). Molecular profiling of prostate cancer.. Curr Urol Rep.

[3] Bettuzzi S, Davalli P, Astancolle S, Carani C, Madeo B (2000). Tumor progression is accompanied by significant changes in the levels of expression of polyamine metabolism regulatory genes and Clusterin (Sulfated Glycoprotein 2) in human prostate cancer specimens.. Cancer Res.

[4] Bettuzzi S, Scaltriti M, Caporali A, Brausi M, D'Arca D (2003). Successful prediction of prostate cancer recurrence by gene profiling in combination with clinical data: a 5-year follow-up study.. Cancer Res.

[5] Konishi H, Steinbach G, Terry N, Lee J, Dubin J (1996). Histone H3 messenger RNA in situ hybridization correlates with in vivo bromodeoxyuridine labeling of S-phase cells in rat colonic epithelium.. Cancer Res.

[6] Del Sal G, Ruaro M, Philipson L, Schneider C (1992). The growth arrest-specific gene, GAS1, is involved in growth suppression.. Cell.

[7] Bettuzzi S, Hiipakka R, Gilna P, Liao S (1989). Identification of an androgen-repressed mRNA in rat ventral prostate as coding for sulphated glycoprotein 2 by cDNA cloning and sequence analysis.. Biochem J.

[8] Lakins J, Bennett SA, Chen JH, Arnold JM, Morrissey C (1998). Clusterin biogenesis is altered during apoptosis in the regressing rat ventral prostate.. J Biol Chem.

[9] Miyake H, Nelson C, Rennie PS, Gleave ME (2000). Testosterone-repressed prostate message-2 is an antiapoptotic gene involved in progression to androgen independence in prostate cancer.. Cancer Res.

[10] July LV, Akbari M, Zellweger T, Jones EC, Goldenberg SL (2002). Clusterin expression is significantly enhanced in prostate cancer cells following androgen withdrawal therapy.. Prostate.

[11] Miyake H, Hara I, Fujisawa M, Gleave ME (2006). The potential of clusterin inhibiting antisense oligodeoxynucleotide therapy for prostate cancer.. Exp Opin Invest Drugs.

[12] Bettuzzi S, Davalli P, Astancolle S, Carani C, Madeo B (2000). Tumor progression is accompanied by significant changes in the levels of expression of polyamine metabolism regulatory genes and clusterin (sulfated glycoprotein 2) in human prostate cancer specimens.. Cancer Res.

[13] Scaltriti M, Brausi M, Amorosi A, Caporali A, D'Arca D (2004). Clusterin (SGP-2, ApoJ) expression is downregulated in low- and high-grade human prostate cancer.. Int J Cancer.

[14] Caporali A, Davalli P, Astancolle S, D'Arca D, Brausi M (2004). The chemopreventive action of catechins in the TRAMP mouse model of prostate carcinogenesis is accompanied by Clusterin over-expression.. Carcinogenesis.

[15] Pins MR, Fiadjoe JE, Korley F, Wong M, Rademaker AW (2004). Clusterin as a possible predictor for biochemical recurrence of prostate cancer following radical prostatectomy with intermediate Gleason scores: a preliminary report.. Prostate Cancer Prostatic Dis.

[16] Bettuzzi S (2003). The new anti-oncogene clusterin and the molecular profiling of prostate cancer progression and prognosis.. Acta Biomedica Ateneo Parmense.

[17] Bettuzzi S, Rizzi F, Belloni L (2007). Clinical relevance of the inhibitory effect of Green Tea Catechins (GTCs) on prostate cancer progression in combination with molecular profiling of catechin-resistant tumors: an integrated view.. Pol J Vet Sci.

[18] Rhodes D, Barrette T, Rubin M, Ghosh D, Chinnaiyan A (2002). Meta-analysis of microarrays: interstudy validation of gene expression profiles reveals pathway dysregulation in prostate cancer.. Cancer Res.

[19] Scaltriti M, Belloni L, Caporali A, Davalli P, Remondini D (2006). Molecular classification of green tea catechin-sensitive and green tea catechin-resistant prostate cancer in the TRAMP mice model by quantitative real-time PCR gene profiling.. Carcinogenesis.

[20] Luo J, Zha S, Gage WR, Dunn TA, Hicks JL (2002). Alpha-methylacyl-CoA racemase: a new molecular marker for prostate cancer.. Cancer Res.

[21] Rubin MA, Zhou M, Dhanasekaran SM, Varambally S, Barrette TR (2002). Alpha-Methylacyl coenzyme A racemase as a tissue biomarker for prostate cancer.. JAMA.

[22] Tinzl M, Marberger M, Horvath S, Chypre C (2004). DD3PCA3 RNA analysis in urine a new perspective for detecting prostate cancer.. Eur Urol.

[23] Pegg AE, Williams-Ashman HG (1968). Biosynthesis of putrescine in the prostate gland of the rat.. Biochem J.

[24] Landers KA, Burger MJ, Tebay MA, Purdie DM, Scells B (2006). Use of multiple biomarkers for a molecular diagnosis of prostate cancer.. Int J Cancer.

[25] Schmidt U, Fuessel S, Koch R, Baretton GB, Lohse A (2006). Quantitative multi-gene expression profiling of primary prostate cancer.. Prostate.

[26] Pfaffl MW, Horgan GW, Dempfle L (2002). Relative Expression Software Tool (*REST©)* for group wise comparison and statistical analysis of relative expression results in real-time PCR.. Nucleic Acids Research.

[27] Glinsky GV, Berezovska O, Glinskii AB (2005). Microarray analysis identifies a death-from-cancer signature predicting therapy failure in patients with multiple types of cancer.. J Clin Invest.

[28] True L, Coleman I, Hawley S, Huang CY, Gifford D (2006). A molecular correlate to the Gleason grading system for prostate adenocarcinoma.. Proc Natl Acad Sci.

[29] Zhang XW, Yap YL, Wei D, Chen F, Danchin A (2005). Molecular diagnosis of human cancer type by gene expression profiles and independent component analysis.. Eur J Hum Genet.

[30] Hessels D, Klein Gunnewiek JM, van Oort I, Karthaus HF, van Leenders GJ (2003). DD3(PCA3)-based molecular urine analysis for the diagnosis of prostate cancer.. Eur Urol.

[31] Rhodes JB, Williams-Ashman HG (1964). Observations on polyamines in male accessory glands of reproduction.. Int J Exp Med.

[32] Williams-Ashman HG, Pegg AE, Lockwood DH (1969). Mechanisms and regulation of polyamine and putrescine biosynthesis in male genital glands and other tissues of mammals.. Adv Enzyme Regul.

[33] Sheth AR, Moodbidri SB (1977). Significance of polyamines in reproduction.. Adv Sex Horm Res.

[34] Fair WR, Wehner N, Brorsson U (1975). Urinary polyamine levels in the diagnosis of carcinoma of the prostate.. J Urol.

[35] Dudoit S, Fridlyand J, Speed T (2002). Comparison of discrimination methods for the classification of tumors using gene expression data.. J Am Stat Ass.

[36] Li T, Zhang C, Ogihara M (2004). A comparative study of feature selection and multiclass classification methods for tissue classification based on gene expression.. Bioinformatics.

[37] Hastie T, Tibshirani R, Friedman J (2001). The Elements of Statistical Learning.

[38] Schaefera KL, Eisenacherb M, Brauna Y, Brachwitza K, Waic DH (2008). Microarray analysis of Ewing's sarcoma family of tumours reveals characteristic gene expression signatures associated with metastasis and resistance to chemotherapy.. Eur J Cancer.

[39] Barrier A, Lemoine A, Boelle PY, Tse C, Brault D (2005). Colon cancer prognosis prediction by gene expression profiling.. Oncogene.

[40] Yu YP, Landsittel D, Jing L, Nelson J, Ren B (2004). Gene expression alterations in prostate cancer predicting tumor aggression and preceding development of malignancy.. J Clin Oncol.

[41] Mehrotra J, Varde S, Wang H, Chiu H, Vargo J (2008). Quantitative, spatial resolution of the epigenetic field effect in prostate cancer.. Prostate.

[42] Uetsuki H, Tsunemori H, Taoka R, Haba R, Ishikawa M (2005). Expression of a novel biomarker, EPCA, in adenocarcinomas and precancerous lesions in the prostate.. J Urol.

[43] Olapade-Olaopa EO, MacKay EH, Taub NA, Sandhu DP, Terry TR (1999). Malignant transformation of human prostatic epithelium is associated with the loss of androgen receptor immunoreactivity in the surrounding stroma.. Clin Cancer Res.

[44] Chandran UR, Dhir R, Ma C, Michalopoulos G, Becich M (2005). Differences in gene expression in prostate cancer, normal appearing prostate tissue adjacent to cancer and prostate tissue from cancer free organ donors.. BMC Cancer.

[45] Scattoni V (2006). Systematic prostate biopsies are more and more often becoming saturation biopsies.. Eur Urol.

[46] Chrouser KL, Lieber MM (2004). Extended and saturation needle biopsy for the diagnosis of prostate cancer.. Curr Urol Rep.

[47] Cheng L, Paterson RF, Beck SD, Parks J (2004). Prostatic intraepithelial neoplasia: an update.. Clin Prostate Cancer.

[48] Sakr W, Partin AW (2001). Histological markers of risk and the role of high-grade prostatic intraepithelial neoplasia.. Urology.

[49] Crissman JD, Sakr WA, Hussein ME, Pontes JE (1993). DNA quantitation of intraepithelial neoplasia and invasive carcinoma of the prostate.. Prostate.

[50] Astancolle S, Guidetti G, Pinna C, Corti A, Bettuzzi S (2000). Increased levels of clusterin (SGP-2) mRNA and protein accompany rat ventral prostate involution following finasteride treatment.. J Endocrinology.

[51] Crescioli C, Ferruzzi P, Caporali A, Mancina R, Comerci A (2003). Inhibition of spontaneous and androgen-induced prostate growth by a nonhypercalcemic calcitriol analog.. Endocrinology.

[52] Crescioli C, Ferruzzi P, Caporali A, Scaltriti M, Bettuzzi S (2004). Inhibition of prostate cell growth by BXL-628, a calcitriol analogue selected for a phase II clinical trial in patients with benign prostate hyperplasia.. Eur J Endocrinol.

[53] Rosenberg ME, Silkensen J (1995). Clusterin: physiologic and pathophysiologic considerations.. Int J Biochem Cell Biol.

[54] Koch-Brandt C, Morgans C (1996). Clusterin: a role in cell survival in the face of apoptosis?. Prog Mol Sub Biol.

[55] Reddy KB, Jin G, Karode MC, Harmony JAK, Howe PH (1996). Transforming growth factor beta (TGF beta)-induced nuclear localization of apolipoprotein J/CLU in epithelial cells.. Biochemistry.

[56] Yang CR, Leskov K, Hosley-Eberlein K, Criswell T, Pink JJ (2000). Nuclear CLU/XIP8, an x-ray-induced Ku70-binding protein that signals cell death.. Proc Natl Acad Sci.

[57] Scaltriti M, Bettuzzi S, Sharrard RM, Caporali A, Caccamo AE (2004). Clusterin overexpression in both malignant and nonmalignant prostate epithelial cells induces cell cycle arrest and apoptosis.. BJU Int.

[58] Caccamo AE, Scaltriti M, Caporali A, D'Arca D, Corti A (2005). Ca2+ depletion caused nuclear translocation of a 45 kDa death-isoform of clusterin and anoikis induction in prostate cells.. Cell Death Diff.

[59] Miyake H, Yamanaka K, Muramaki M, Kurahashi T, Gleave M (2005). Enhanced expression of the secreted form of clusterin following neoadjuvant hormonal therapy as a prognostic predictor in patients undergoing radical prostatectomy for prostate cancer.. Oncol Rep.

[60] Gleave M, Miyake H (2005). Use of antisense oligonucleotides targeting the cytoprotective gene, clusterin, to enhance androgen- and chemo-sensitivity in prostate cancer.. World J Urology.

[61] Lapointe J, Li C, Higgins J, van de Rijn M, Bair E (2004). Gene expression profiling identifies clinically relevant subtypes of prostate cancer.. Proc Natl Acad Sci USA.

[62] Bettuzzi S, Scorcioni F, Astancolle A, Davalli P, Scaltriti M (2002). Clusterin (SGP-2) transient overexpression decreases proliferation rate of SV40-immortalised human prostate epithelial cells by slowing down cell cycle progression.. Oncogene.

[63] Scaltriti M, Santamaria A, Paciucci R, Bettuzzi S (2004). Intracellular clusterin induces G2-M phase arrest and cell death in PC-3 prostate cancer cells.. Cancer Res.

[64] Chen T, Turner J, McCarthy S, Scaltriti M, Bettuzzi S (2004). Clusterin Mediated Apoptosis is Regulated by APC and is P21 Dependent but P53 Independent.. Cancer Res.

[65] Montironi R, Mazzucchelli R, Scarpelli M, Lopez-Beltran A, Fellegara G (2005). Gleason Grading of prostate cancer in needle biopsies or prostatectomy specimens: contemporary approach, current clinical significance and sources of pathology discrepancies.. BJU Int.

